# Supplementary material for: Efficient Propylene/Ethylene Separation in Highly Porous Metal–Organic Frameworks
Source: Materials (Basel). 2022 Dec 23;16(1):154. doi: 10.3390/ma16010154 (PMC9821945; doi:10.3390/ma16010154)
Supplement: Supplementary file 1 [file materials-16-00154-s001.zip › materials-2073577-supplementary.pdf]

materials

Supplementary Material

Efficient propylene/ethylene separation in highly porous metal-organic frameworks

Xiao-Min Liu 1, Lin-Hua Xie 2,* and Yufeng Wu 1,*

1 Institute of Circular Economy, Beijing University of Technology, Beijing 100124, P. R. China
2 Beijing Key Laboratory for Green Catalysis and Separation and Department of Environmental Chemical Engineering, Faculty of Environment and Life, Beijing University of Technology, Beijing 100124, China

Table S1. Crystal data and structure refinements for Fe2M-L.

	Fe_2Mn-L	Fe_2Co-L	Fe_2Ni-L
empirical formula	$Fe_2MnC_{78}H_{112}O_{28}$	$Fe_2CoC_{78}H_{112}O_{28}$	$Fe_2NiC_{78}H_{112}O_{28}$
M	1832.43	1836.42	1836.20
Temperature/K	100(2)	100(2)	100(2)
crystal system	Trigonal	Trigonal	Trigonal
space group	R-3c	R-3c	R-3c
a/Å	27.4975(2)	27.6077(3)	27.5618(2)
c/Å	73.1343(7)	72.6748(11)	72.9933(6)
V/Å^3	47889.2(8)	47970.6(13)	48020.7(8)
Z	18	18	18
D_{calc}/g cm^{-3}	1.144	1.144	1.143
μ/mm^{-1}	3.694	3.943	2.940
reflns collected	54790	55582	58594
Independent reflns	9061	9081	10223
R_{int}	0.0620	0.0686	0.0349
θ range (°)	3.546 to 65.080	3.559 to 65.082	3.549 to 71.075
Completeness	99.7 %	99.8 %	99.8 %
GOF	1.025	1.017	1.019
$R_1[I > 2\sigma(I)]^a$	0.0639	0.0675	0.0592
wR_2(all data)^b	0.1569	0.1615	0.1497
$\Delta\rho_{max/min}$ (e/Å^3)	0.685/-0.651	0.999/-0.701	0.903/-0.445

[a] $R_1 = \sum||F_o| - |F_c||/\sum|F_o|$. [b] $wR_2 = [\sum w(F_o^2 - F_c^2)^2/\sum w(F_o^2)^2]^{1/2}$.

Table S2. ICP-AES analysis results for the bulk samples of Fe2M-L.

Sample	Percentage of Fe ion	Percentage of M ion	Molar ratio of Fe to M
**Fe_2Mn-L**	66.5	33.5	1.99
**Fe_2Co-L**	66.8	33.2	2.01
**Fe_2Ni-L**	66.2	33.8	1.96

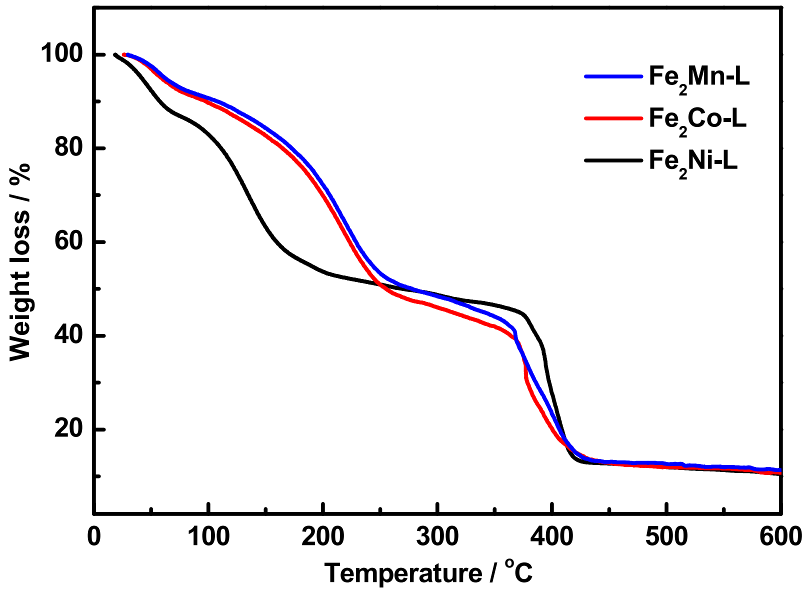

Figure S1. TGA curves of the as-synthesized Fe2M-L samples, indicating the presence of about 12 DMF molecules per formula inside their pores.

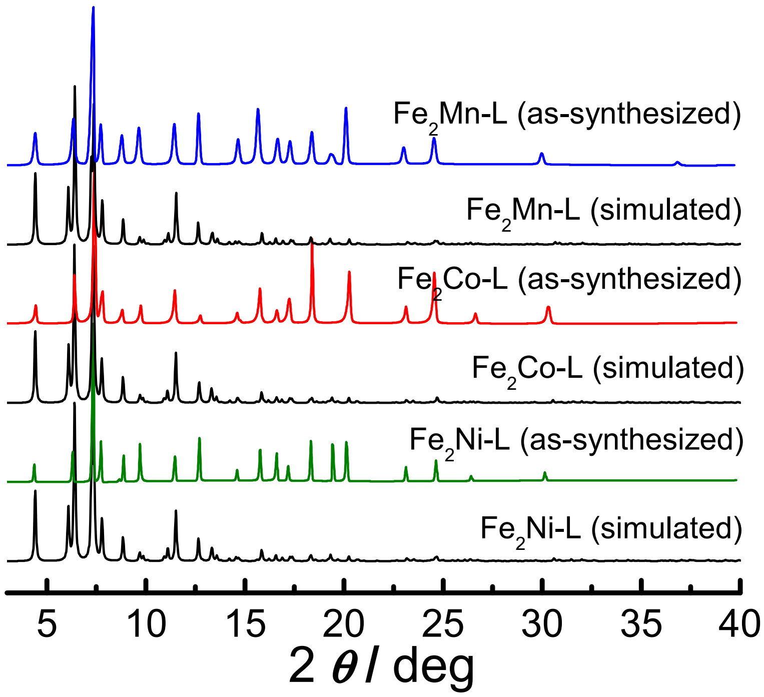

Figure S2. PXRD patterns of the as-synthesized Fe2M-L samples in comparison with their simulated PXRD patterns.

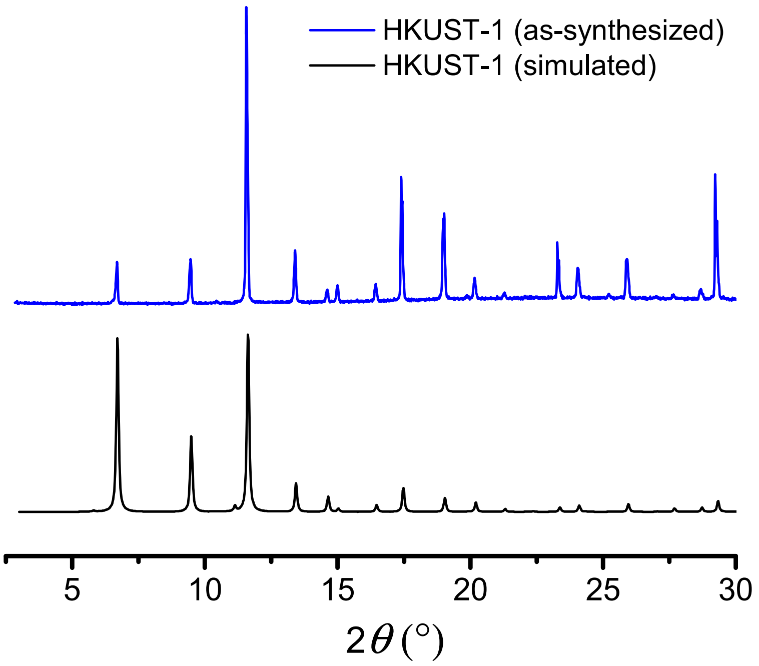

Figure S3. PXRD pattern of the as-synthesized HKUST-1 sample in comparison with its simulated PXRD pattern.

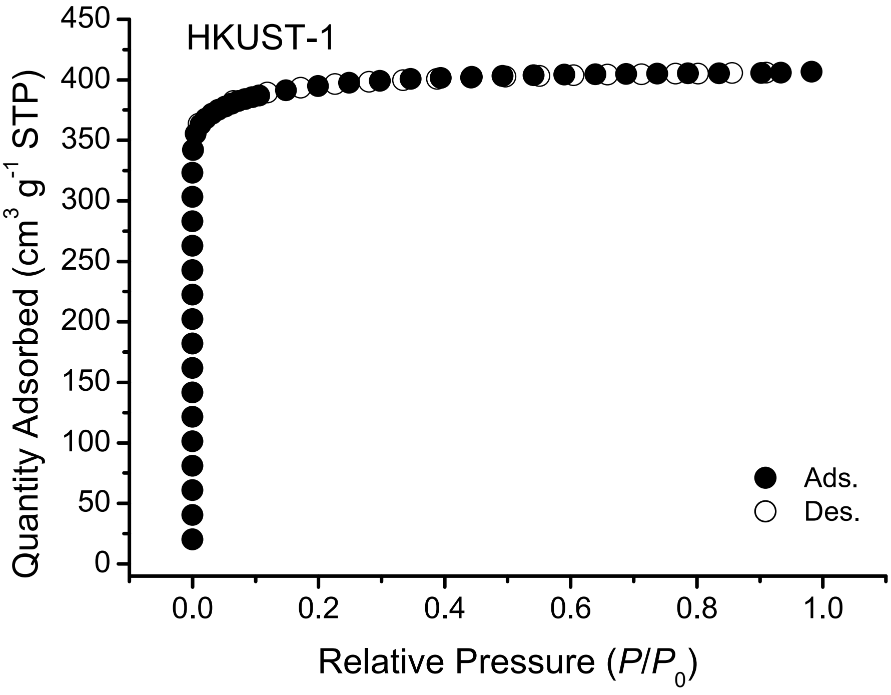

Figure S4. N2 adsorption isotherm of the prepared HKUST-1 sample recorded at 77 K.

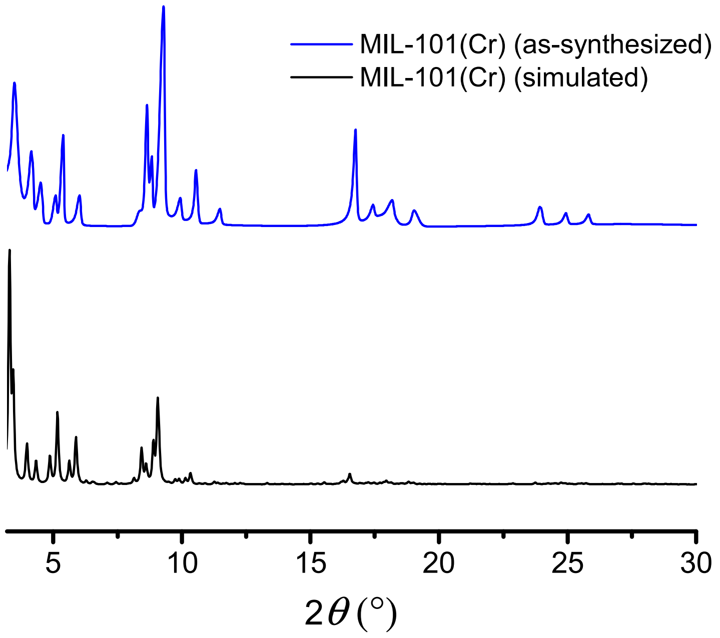

Figure S5. PXRD pattern of the as-synthesized MIL-101(Cr) sample in comparison with its simulated PXRD pattern.

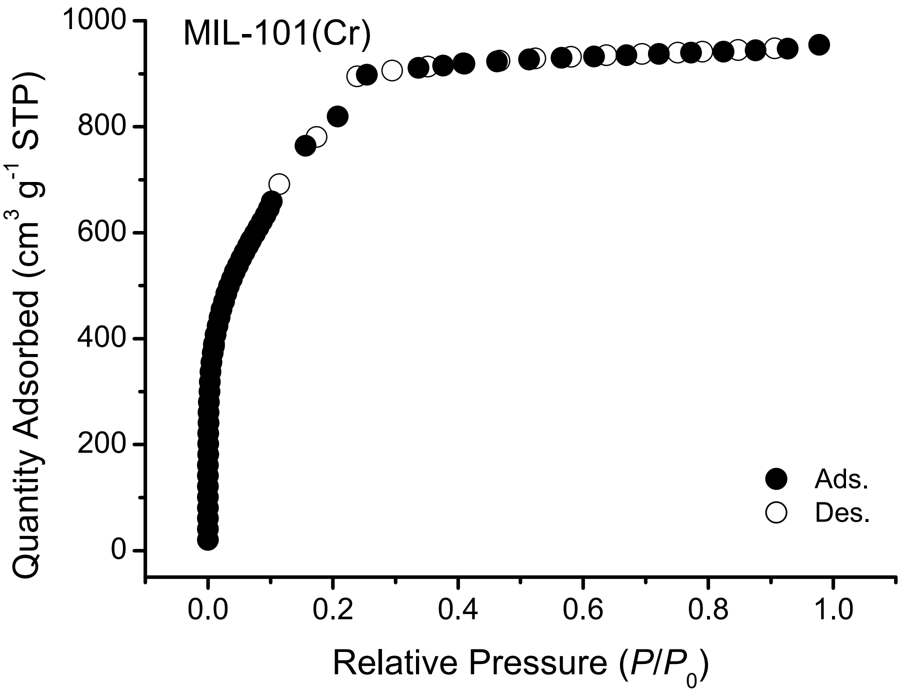

Figure S6. N2 adsorption isotherm of the prepared MIL-101(Cr) sample recorded at 77 K.

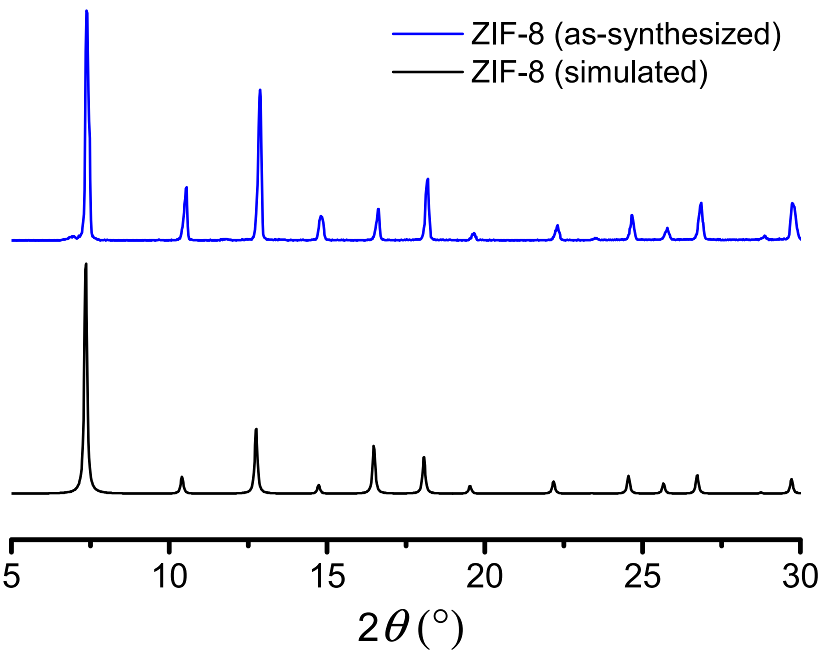

Figure S7. PXRD pattern of the as-synthesized ZIF-8 sample in comparison with its simulated PXRD pattern.

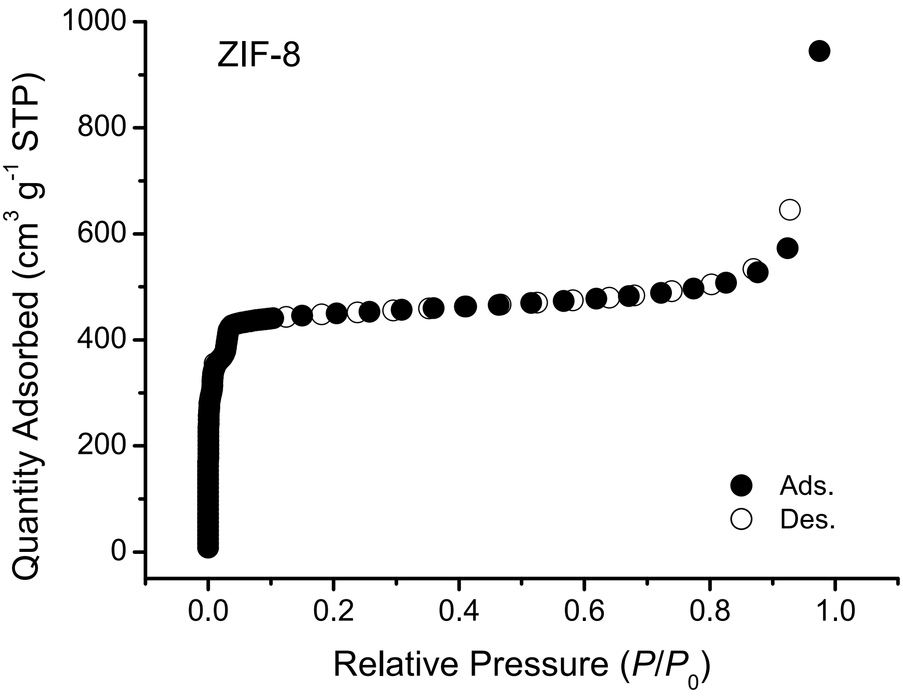

Figure S8. N2 adsorption isotherm of the prepared ZIF-8 sample recorded at 77 K. .

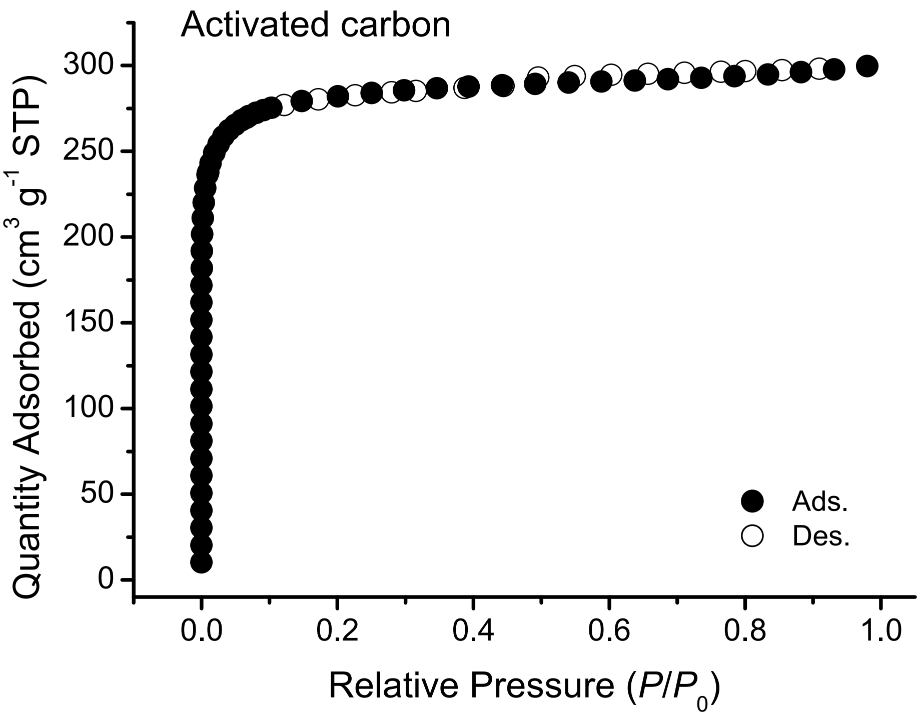

Figure S9. N2 adsorption isotherm of the activated carbon sample recorded at 77 K.

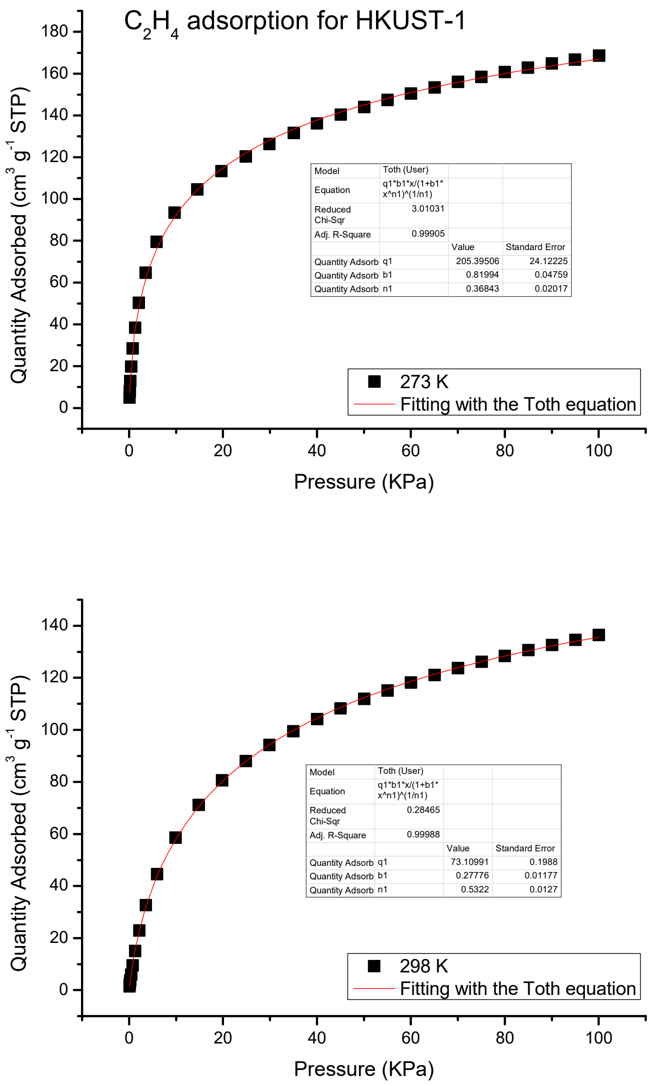

Figure S10. C2H4 adsorption isotherms of the prepared HKUST-1 sample recorded at 273 and 298 K, and fitting results of the isotherms by the Toth equation.

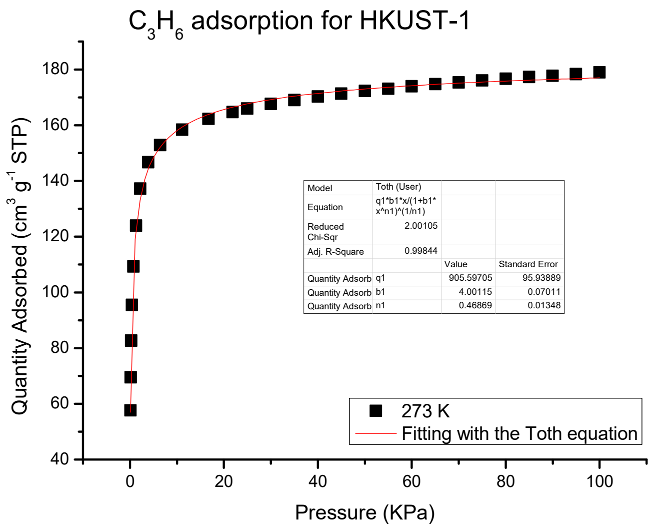

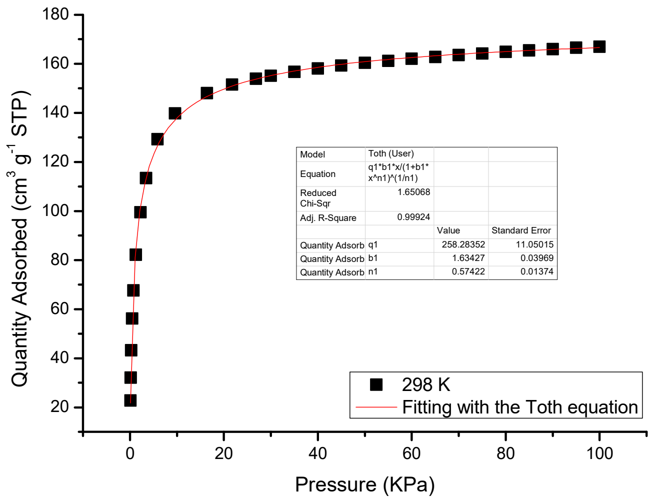

Figure S11. C3H6 adsorption isotherms of the prepared HKUST-1 sample recorded at 273 and 298 K, and fitting results of the isotherms by the Toth equation.

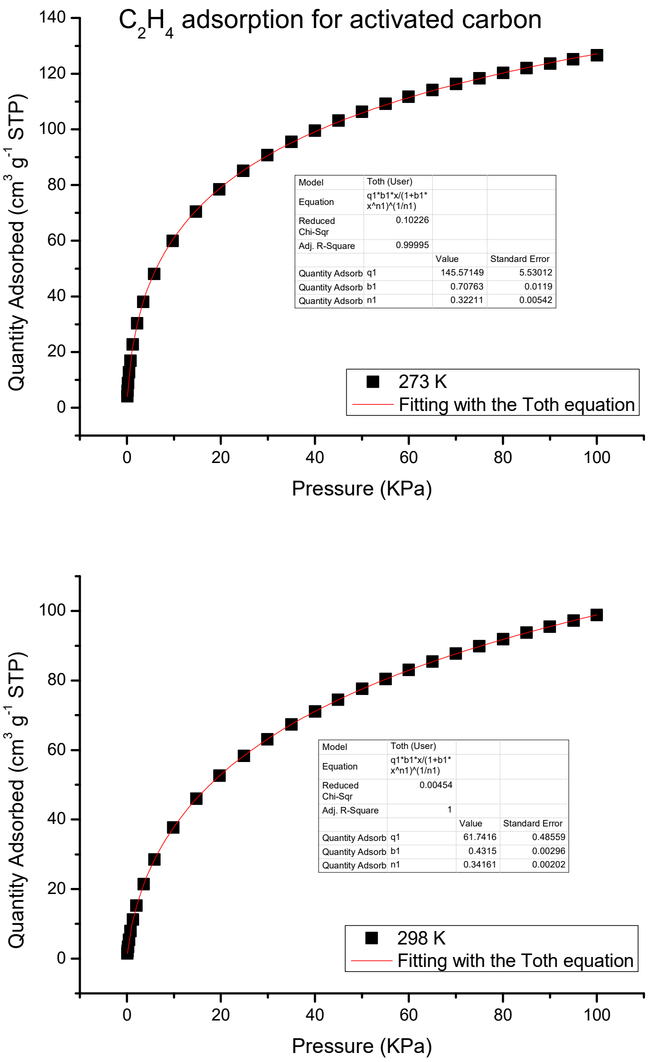

Figure S12. C2H4 adsorption isotherms of the activated carbon sample recorded at 273 and 298 K, 39
and fitting results of the isotherms by the Toth equation. 40

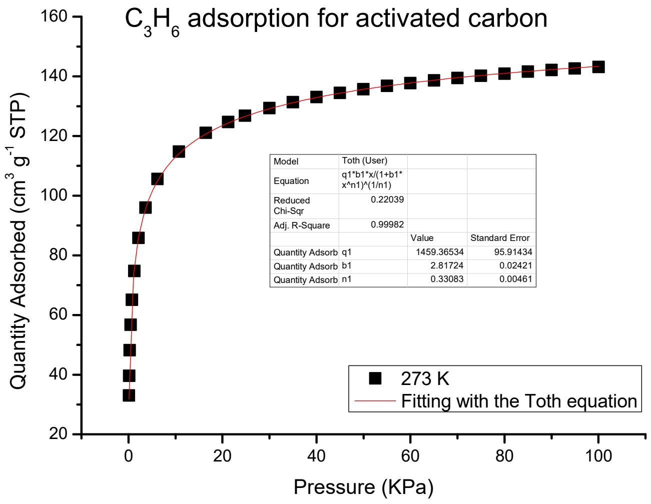

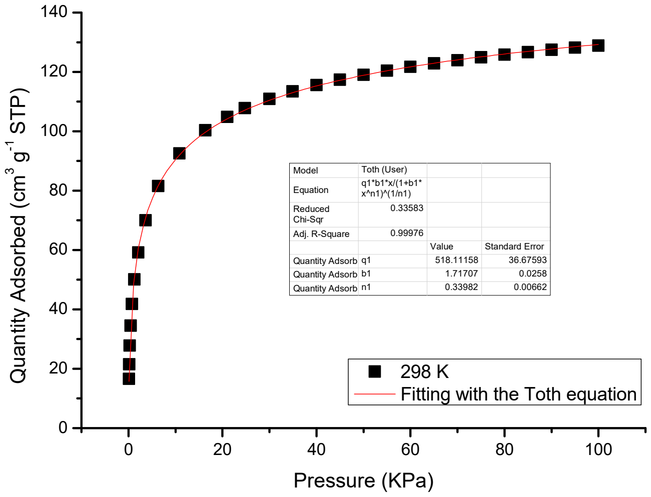

Figure S13. C3H6 adsorption isotherms of the activated carbon sample recorded at 273 and 298 K, and fitting results of the isotherms by the Toth equation.

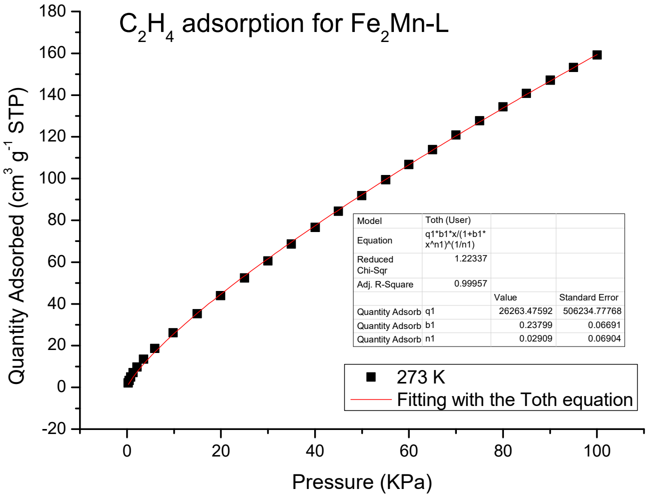

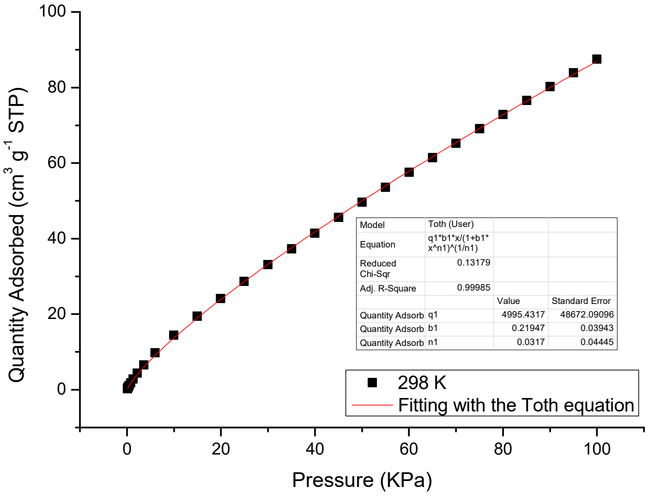

43 **Figure S14.** C2H4 adsorption isotherms of the Fe2Mn-L sample recorded at 273 and 298 K, and
44 fitting results of the isotherms by the Toth equation.

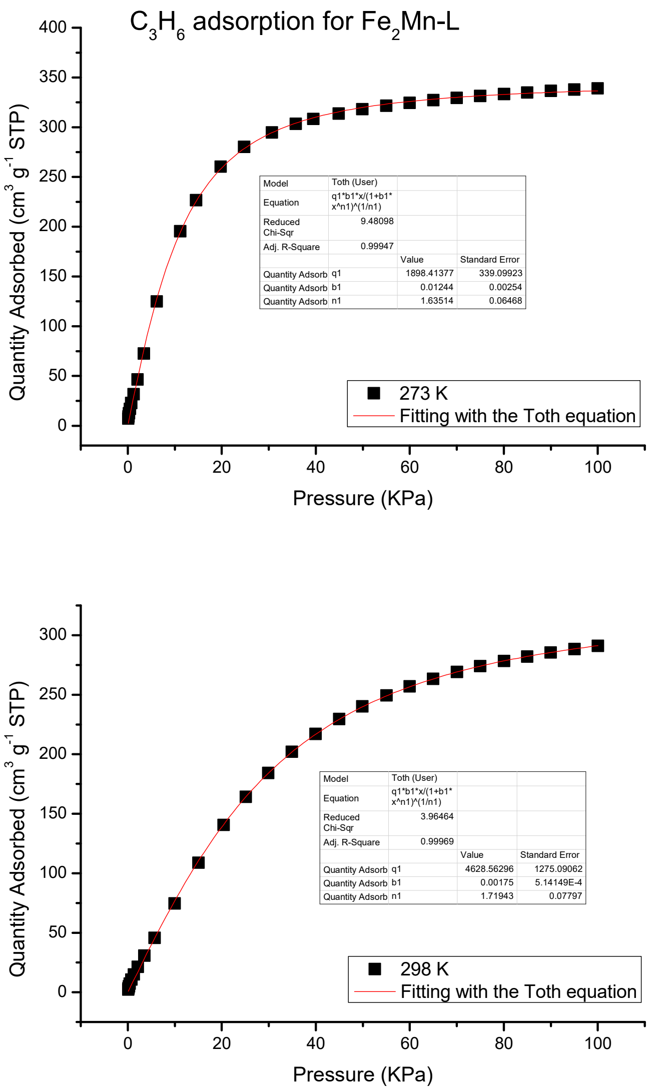

Figure S15. C3H6 adsorption isotherms of the Fe2Mn-L sample recorded at 273 and 298 K, and fitting results of the isotherms by the Toth equation.

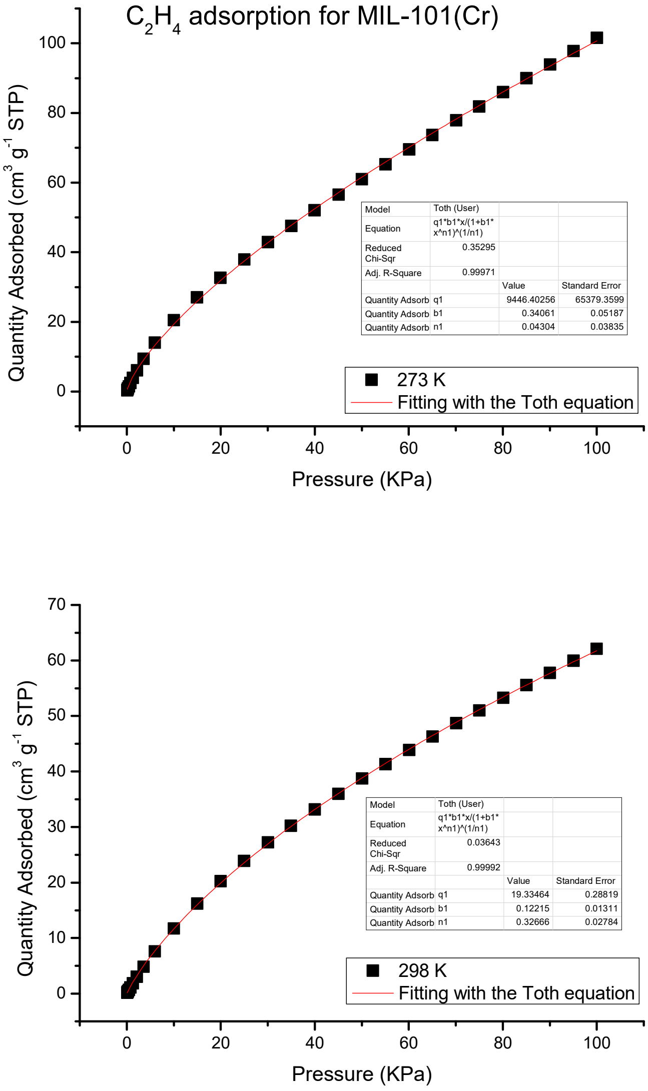

Figure S16. C2H4 adsorption isotherms of the MIL-101(Cr) sample recorded at 273 and 298 K, and fitting results of the isotherms by the Toth equation.

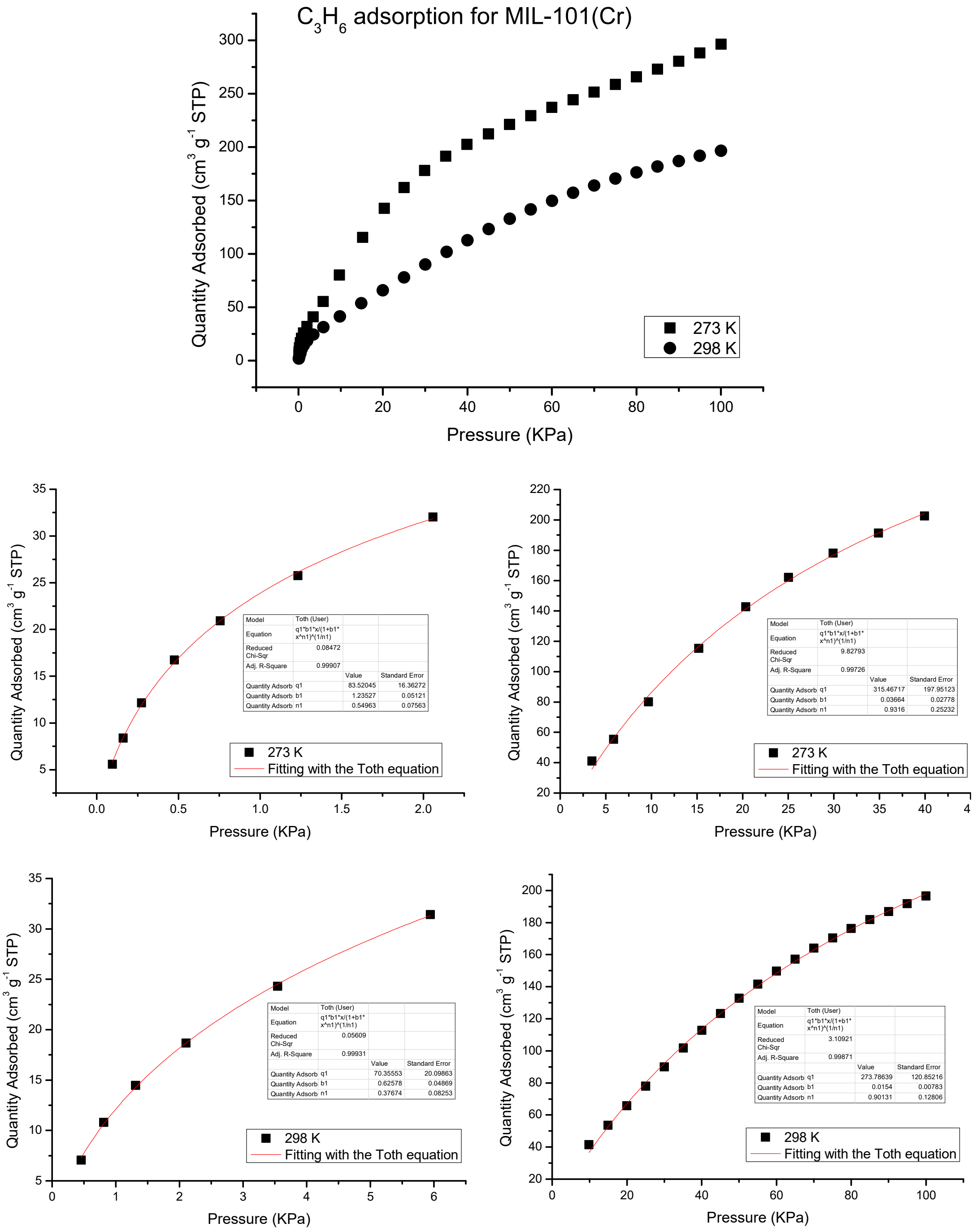

Figure S17. C3H6 adsorption isotherms of the MIL-101(Cr) sample recorded at 273 and 298 K, and fitting results of the isotherms by the Toth equation. To get better fitting results, the data at low pressure range and the data at high pressure range were fitted separately. .

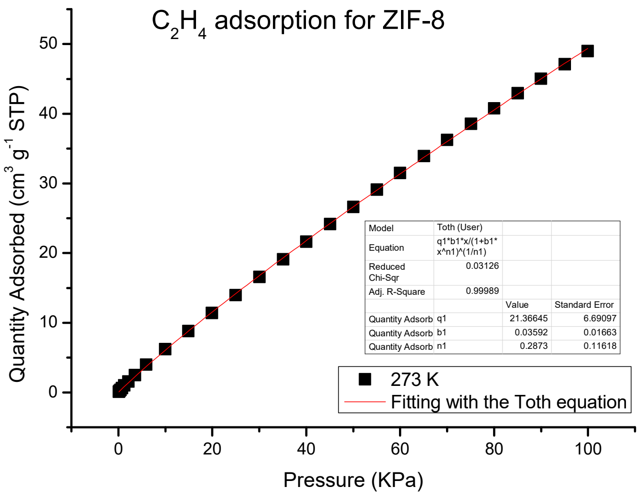

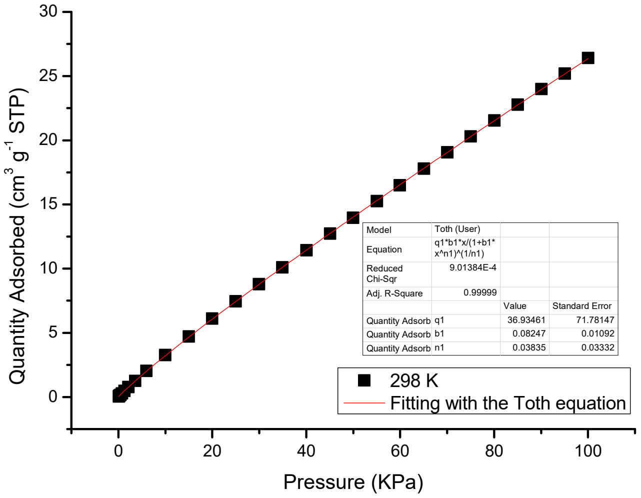

Figure S18. C2H4 adsorption isotherms of the ZIF-8 sample recorded at 273 and 298 K, and fitting results of the isotherms by the Toth equation.

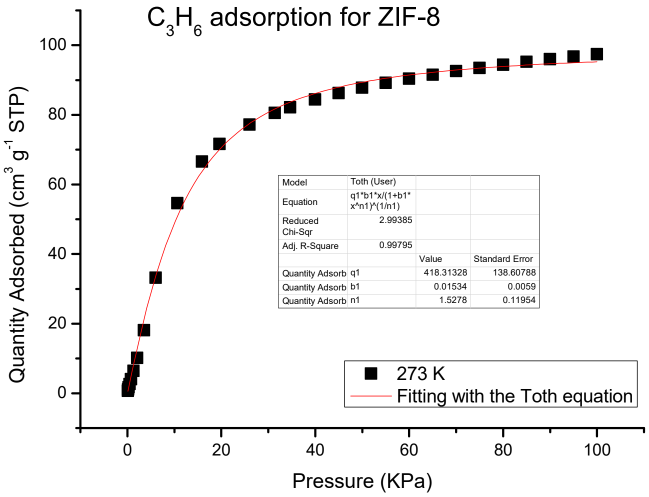

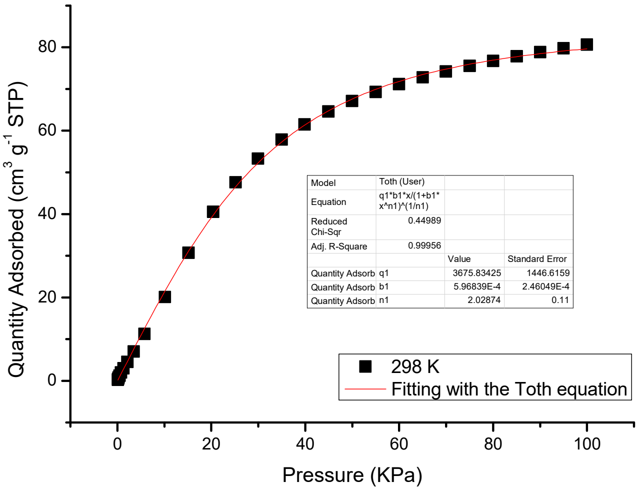

Figure S19. C3H6 adsorption isotherms of the ZIF-8 sample recorded at 273 and 298 K, and fitting results of the isotherms by the Toth equation.

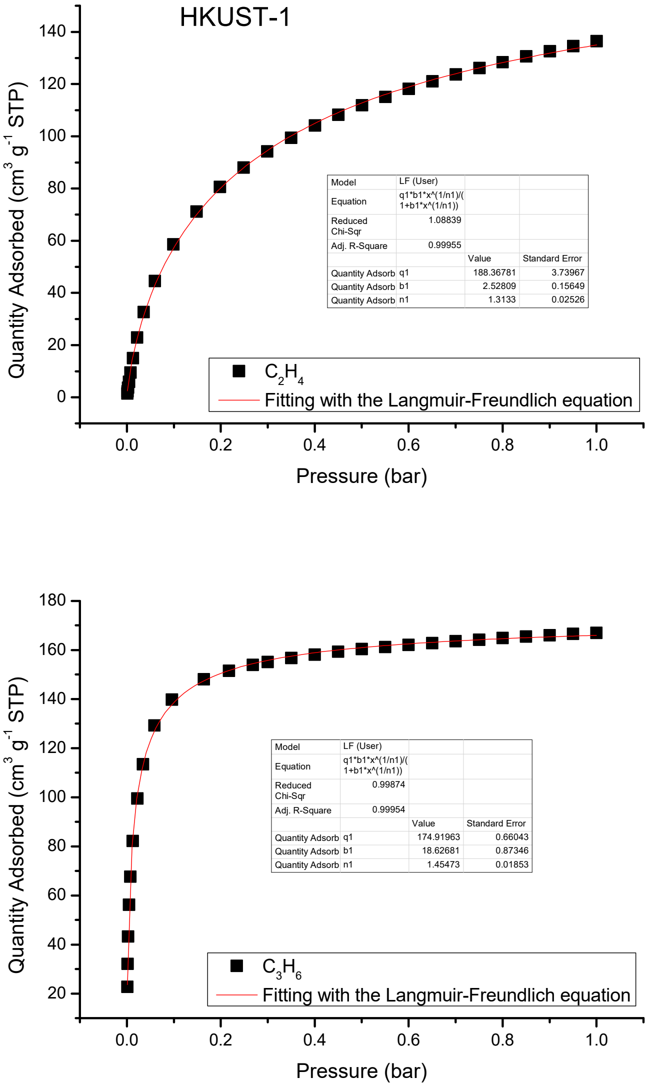

Figure S20. C2H4 and C3H6 adsorption isotherms of the prepared HKUST-1 sample recorded at 298 K, and fitting results of the isotherms by the Langmuir-Freundlich equation.

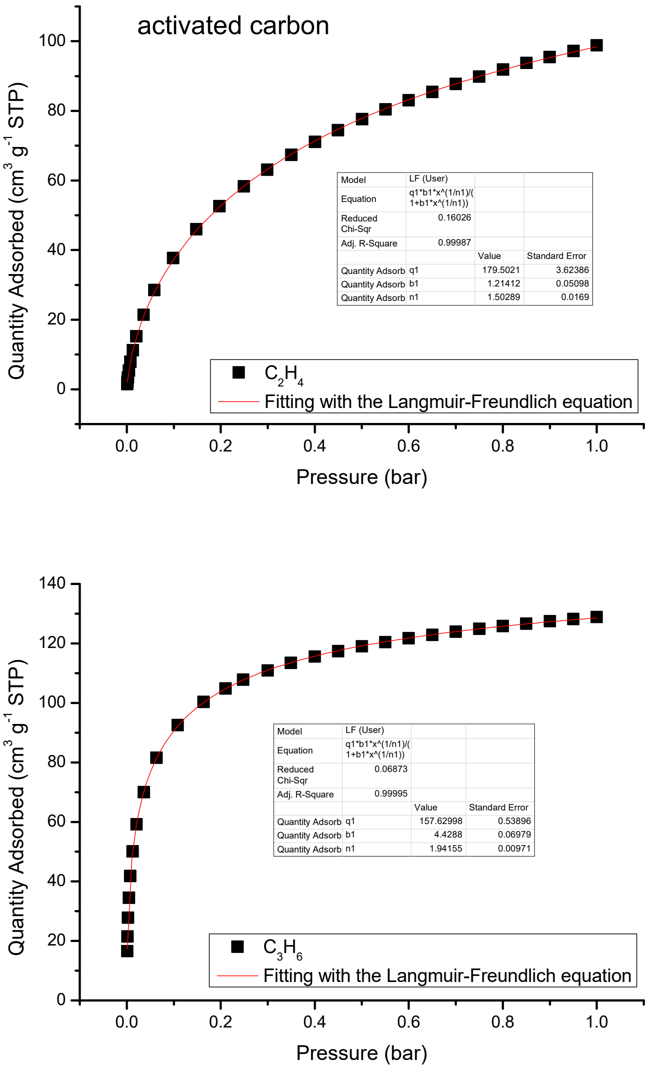

Figure S21. C2H4 and C3H6 adsorption isotherms of the activated carbon sample recorded at 298 K, and fitting results of the isotherms by the Langmuir-Freundlich equation.

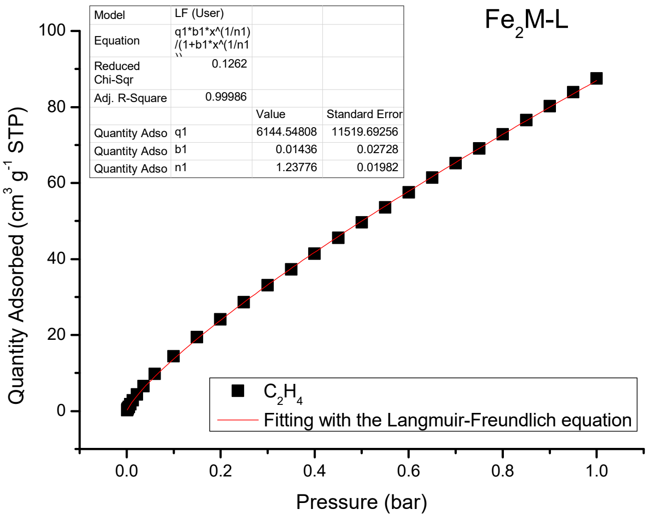

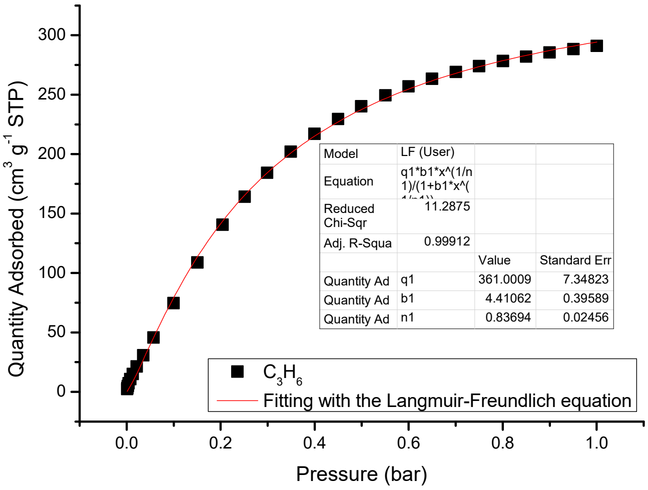

Figure S22. C2H4 and C3H6 adsorption isotherms of the prepared Fe2M-L sample recorded at 298 K, and fitting results of the isotherms by the Langmuir-Freundlich equation.

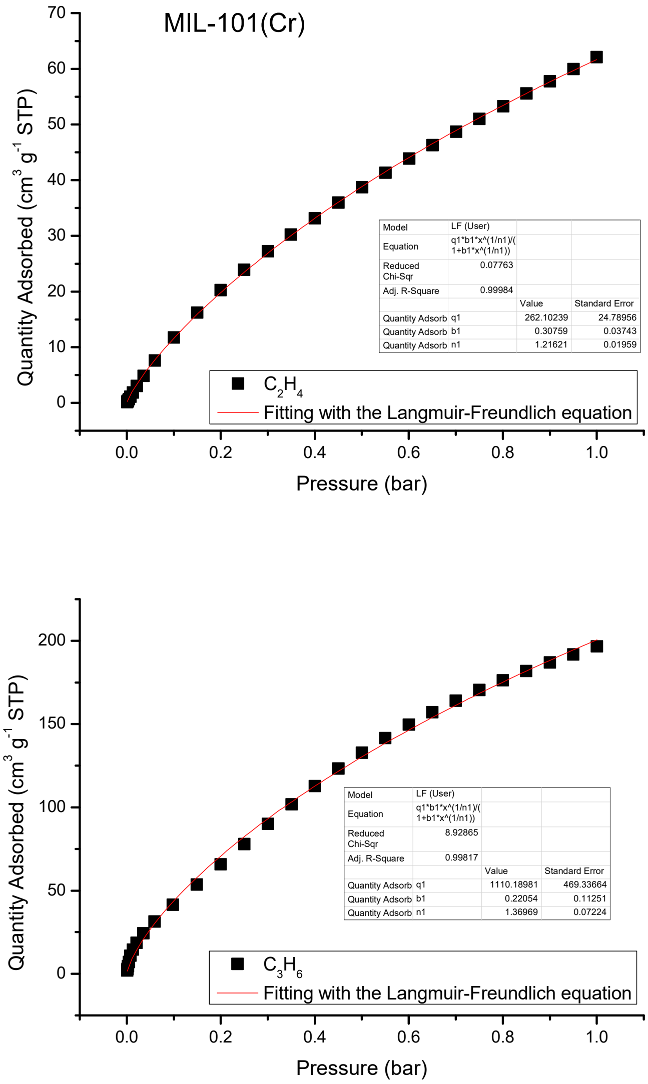

Figure S23. C2H4 and C3H6 adsorption isotherms of the prepared MIL-101(Cr) sample recorded at 298 K, and fitting results of the isotherms by the Langmuir-Freundlich equation.

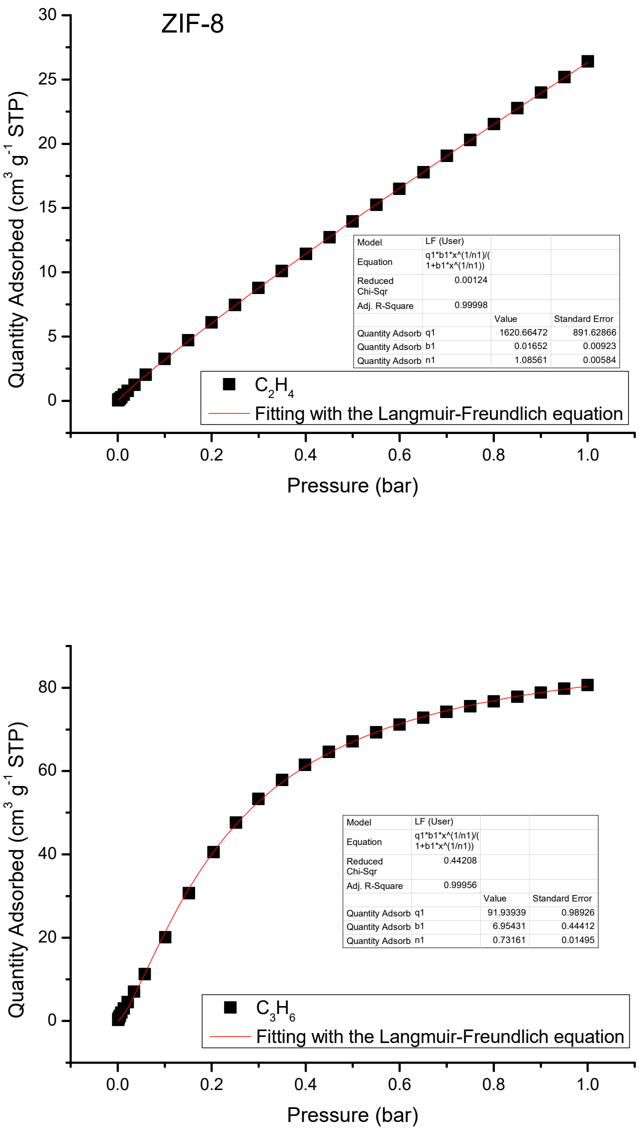

Figure S24. C2H4 and C3H6 adsorption isotherms of the prepared ZIF-8 sample recorded at 298 K, and fitting results of the isotherms by the Langmuir-Freundlich equation.
